# Comparative Study on the Hypoglycemic and Antioxidative Effects of Fermented Paste (Doenjang) Prepared from Soybean and Brown Rice Mixed with Rice Bran or Red Ginseng Marc in Mice Fed with High Fat Diet

**DOI:** 10.3390/nu6104610

**Published:** 2014-10-22

**Authors:** Soo Im Chung, Catherine W. Rico, Mi Young Kang

**Affiliations:** Department of Food Science and Nutrition, Brain Korea 21 Plus, Kyungpook National Universiy, 1370 Sankyuk-ong, Degu 702-701, Korea; E-Mails: zizibe0312@nate.com (S.I.C.); ckwrico@gmail.com (C.W.R.)

**Keywords:** antioxidative effect, brown rice, fermented paste, hypoglycemic effect, soybean

## Abstract

The effects of fermented paste made from soybean, brown rice, or brown rice in combination with rice bran or red ginseng marc on the glucose metabolism and antioxidative defense system in high fat-fed mice were investigated. The mice were given experimental diets for eight weeks: Normal control, high fat, and high fat supplemented with soybean fermented paste, brown rice fermented paste, brown rice-rice bran fermented paste, or brown rice-red ginseng marc fermented paste. The high fat group showed markedly higher blood glucose level and erythrocyte lipid peroxidation than the normal control group. Diet supplementation of fermented paste inhibited the high fat-induced hyperglycemia and oxidative stress via regulation of the glucose-regulating and antioxidant enzymes activities. The soybean and brown rice-red ginseng marc fermented pastes were the most effective in improving the glucose metabolism and antioxidant defense status in mice under high fat diet condition. These findings illustrate that brown rice, in combination with red ginseng marc, may be useful in the development of fermented paste with strong hypoglycemic and antioxidative activities.

## 1. Introduction

Fermented soybean paste is commonly used as a dipping condiment or flavor seasoning in various soups and stews in Korea. In 2009, this Korean soybean paste was registered in CODEX Alimentarius, a collection of international food standards and guidelines, and became an internationally accepted food [1]. For the past years, its popularity has increased due to its distinct flavor and high nutritional value. It is rich in essential amino acids, minerals, and vitamins [2]. Furthermore, studies have shown that fermented paste and other fermented soybean products contain higher protein and phenolic contents, stronger antioxidant effect, and greater antimutagenic and anticancer activities than raw and cooked soybeans [3,4,5].

Rice, the second most widely grown cereal crop in the world, is mostly produced and consumed in Asia. However, the consumption of rice is steadily declining in Asian countries, particularly in Korea, due to the changing dietary habits. To increase rice consumption, alternative ways of using rice in food preparations are continuously being explored by scientists and researchers. As a result, rice-based value-added products, such as rice flour, breakfast cereals, rice noodles, and rice milk, have been developed. Rice bran is a by-product of the rice milling industry and commonly used as animal feed. It has various health-promoting properties including hypolipidemic, hypoglycemic, and antioxidative activities [6,7,8]. Red ginseng marc, on the other hand, is a by-product of ginseng extract production. It is often discarded as waste but studies have shown that it possesses pharmacological properties like immunomodulatory and anti-cancer effects [9,10].

In the present study, fermented paste was prepared using brown rice, instead of soybean, as the main component. With the reported functional properties of rice bran and red ginseng marc, a fermented paste using brown rice in combination with these by-products was also prepared. A number of studies revealed that fermented brown rice and rice bran have health beneficial effects, including anti-tumor and anti-cancer activities [11,12,13]. Moreover, fermented red ginseng marc has been shown to have strong antioxidant effect [14]. The development of rice fermented paste with added rice bran or red ginseng marc may not only increase the market value and consumption of rice, but it may also increase the utilization of rice bran and red ginseng marc and provide health beneficial effects that are comparable with that of the soybean fermented paste. It is therefore essential to have knowledge and understanding on the physiological and metabolic activities of fermented rice paste. A previous investigation on fermented paste made from brown rice revealed that it could suppress body weight gain and improve the lipid profile in high fat diet-fed mice and these physiological effects were further enhanced with the inclusion of rice bran or red ginseng marc [15].

Fermented soybean products have been reported to exhibit antidiabetic effects and antioxidant activity [1,16]. However, studies on fermented paste in relation to glucose metabolism and antioxidative properties are limited. With the growing global problem of diabetes and hyperglycemia, there is an increasing demand for natural products and functional foods with hypoglycemic activity and strong antioxidant property. This study was carried out to investigate the comparative effects of fermented paste prepared from fermented soybean, brown rice, and brown rice with added rice bran or red ginseng marc on the glucose metabolism and antioxidative defense system in mice under a high fat diet condition.

## 2. Experimental Section

### 2.1. Materials and Chemicals

The soybean (SB) fermented paste and brown rice were purchased from a local market in Daegu, Korea. The rice bran was provided by Rice Processing Complex (RPC, Gimcheon, Korea), while the red ginseng marc was obtained from Punggi Ginseng Farming Corporation (YEongju, Gyeongbuk, Korea). The *Aspergillus oryzae* was procured from NUC Electronics Co., Ltd. (Daegu, Korea). The chemicals such as ethanol, ketamine-HCl, potassium phosphate buffer, trichloroacetic acid, and thiobarbituric acid were purchased from Merck KGaA (Darmstadt, Germany). All other chemicals used were obtained from Sigma-Aldrich, Inc. (Steinhein, Germany).

### 2.2. Preparation of Brown Rice Fermented Paste Samples

The brown rice (BR) fermented paste was prepared according to the manufacturing process for commercial fermented paste with some modifications [17]. Briefly, the rice grains were washed, soaked in water for 3 h at 4 °C, and cooked using an electric rice cooker. After cooling, the cooked rice was inoculated with *A. oryzae* (0.2%, w/w) and incubated for 3 days at 30 °C. Salt was added to the resulting koji (12%, w/w), mixed using a food processor, and fermented and ripened for 30 days at 30 °C. The same method was used for the preparation of the brown rice-rice bran (BRB) and brown rice-red ginseng marc (BRG) fermented paste samples. The rice bran and red ginseng marc were washed and steamed separately for 40 min. Prior to inoculation with *A. oryzae*, the cooked brown rice was added with rice bran (20%, w/w) or red ginseng (30%, w/w). All fermented paste samples were freeze-dried at −70 °C prior to use. Their proximate compositions are presented in Table 1.

**Table 1:** Proximate composition (% dry basis) of the fermented paste samples.

Composition	Fermented Paste ^(1)^			
	SB	BR	BRB	BRG
Moisture	4.52	4.91	4.96	5.23
Carbohydrate	45.55	74.72	68.77	70.79
Crude protein	23.42	7.82	9.45	8.15
Crude fat	0.91	0.41	1.13	0.16
Dietary fiber	4.36	2.47	4.16	3.04
Ash	21.24	9.67	11.53	12.63

^(1)^ SB, soybean fermented paste; BR, brown rice fermented paste; BRB, brown rice and rice bran fermented paste; BRG, brown rice and red ginseng marc fermented paste.

### 2.3. Animals and Diets

Forty-eight male C57BL/6N mice (4-week-old), weighing 12 g, were obtained from Orient Inc. (Seoul, Korea). Each mouse was housed in a stainless steel cage in a room maintained at 25 °C with 50% relative humidity and 12/12 h light/dark cycle. Upon arrival, the animals were fed with pelletized chow diet for 2 weeks. They were then randomly divided into 6 dietary groups (*n* = 8). The first and second groups were fed with normal control (NC group) and high fat (20%, w/w, HF group) diets, respectively. The other 4 groups were fed with a high fat diet supplemented with soybean fermented paste (HF + SB group), brown rice fermented paste (HF + BR group), brown rice-rice bran fermented paste (HF + BRB group), or brown rice-red ginseng marc fermented paste (HF + BRG group). The composition of the experimental diet (Table 2) was based on the AIN-76 semisynthetic diet [18]. The diets for all animal groups, except for the NC group, were added with 17% (w/w) lard to make the diet high fat. The mice were fed for eight weeks and allowed free access to food and water. The feed intake and weight gain were measured daily and weekly, respectively. At the end of the experimental period, the mice were anaesthetized with ketamine-HCl following a 12-h fast. The blood samples were drawn from the inferior vena cava into a heparin-coated tube and centrifuged at 1000× *g* for 15 min at 4 °C to obtain the plasma and erythrocyte. The plasma and buffy coat were removed after centrifugation and the erythrocytes were washed with physiological saline, followed by hemolysis with distilled water [19]. The concentration of hemoglobin was determined using a commercial assay kit (Asan Pharmaceutical, Seoul, Korea). The liver and adipose tissues (epididymal, perirenal, and inguinal) were removed, rinsed with physiological saline, weighed, and stored at −70 °C until analysis. The current study protocol was approved by the Ethics Committee of Kyungpook National University for animal studies.

**Table 2:** Composition of the experimental diets (% w/w).

Component	NC ^(1)^	HF	HF + SB	HF + BR	HF + BRB	HF + BRG
Casein	20	20	17.58	19.12	18.96	19.09
dl-Methionine	0.3	0.3	0.3	0.3	0.3	0.3
Sucrose	50	50	45.24	42.32	42.91	42.70
Corn starch	15	-	-	-	-	-
Cellulose	5	5	4.54	4.73	4.56	4.67
Corn oil	5	3	3	3	3	3
Cholinbitartrate	0.2	0.2	0.2	0.2	0.2	0.2
Mineral mixture ^(2)^	3.5	3.5	1.37	2.52	2.34	2.23
Vitamin mixture ^(3)^	1	1	1	1	1	1
Lard	-	17	16.83	16.88	16.80	16.90
SB	-	-	10	-	-	-
BR	-	-	-	10	-	-
BRB	-	-	-	-	10	-
BRG	-	-	-	-	-	10
Total (%)	100	100	100	100	100	100

^(1)^ NC, normal control diet; HF, high fat diet; HF + SB, high fat diet + soybean fermented paste; HF + BR, high fat diet + brown rice fermented paste; HF + BRB, high fat diet + brown rice and rice bran fermented paste; HF + BRG, high fat diet + brown rice and red ginseng marc fermented paste; ^(2)^ AIN-76 mineral mixture; ^(3)^ AIN-76 vitamin mixture.

### 2.4. Determination of Blood Glucose Level

The blood glucose level was measured using Accu-Chek Active Blood Glucose Test Strips (Roche Diagnostics GmbH, Germany). The blood samples were drawn from the tail vein of the mice at 2-week intervals for 8 weeks.

### 2.5. Determination of Hepatic Glycogen and Plasma Insulin Levels

The concentration of glycogen was determined according to the method described by Seifter *et al.* [20]. A 100 mg of fresh liver was mixed with 30% KOH and heated at 100 °C for 30 min. The mixture was added with 1.5 mL ethanol (95%) and kept overnight at 4 °C. The pellet was then mixed with 4 mL distilled water. The mixture (500 µL) was added with 0.2% anthrone (in 95% H_2_SO_4_) and its absorbance was measured at 620 nm. The results were calculated on the basis of a standard calibration curve of glucose. The insulin concentration was measured using enzyme-linked immunosorbent assay (ELISA) kits (TMB Mouse Insulin ELISA kit, Sibayagi, Japan).

### 2.6. Determination of Lipid Peroxidation

The plasma and erythrocyte thiobarbituric acid reactive substances (TBARS) were measured using the method of Ohkawa *et al.* [21]. A 50 µL of the plasma and red blood cell preparation was added with trichloroacetic acid (5%, v/v) and 0.06 M thiobarbituric and incubated at 80 °C for 90 min. The mixtures were cooled at room temperature and centrifuged at 2000 rpm for 25 min. The absorbance of the resulting supernatant was determined at 535 nm. A malondialdehyde (MDA) solution was used as standard and the results were calculated and expressed as nmol MDA/mL plasma or g Hb.

### 2.7. Measurement of Hepatic Glucose-Regulating Enzyme and Antioxidant Enzyme Activities

The hepatic enzyme source was prepared based on the method of Hulcher and Oleson [22]. A 0.3 g liver was homogenized in buffer solution (0.1 M triethanolamine, 0.2 M EDTA, and 0.002 M dithiothreitol) and centrifuged at 1000× *g* for 15 min at 4 °C. The supernatant was centrifuged at 10,000× *g* for 15 min at 4 °C and the resulting precipitate served as the mitochondrial fraction, while the supernatant was further centrifuged at 105,000× *g* for 1 h at 4 °C. The resulting supernatant and precipitate were the cytosol and microsome fractions, respectively.

The glucokinase (GK) activity was measured according to the method described by Davidson and Arion [23] with slight modifications. A 0.98 mL of the reaction mixture (50 mM Hepes-NaOH (pH 7.4), 100 mM KCl, 7.5 mM MgCl_2_, 2.5 mM dithioerythritol, 10 mg/mL albumin, 10 mM glucose, 4 units of glucose-6-phosphate dehydrogenase, 50 mM NAD^+^, and 10 µL cytosol) was pre-incubated at 37 °C for 10 min. The reaction was initiated with the addition of 10 µL of 5 mM ATP and the mixture was incubated at 37 °C for 10 min. The change in absorbance at 340 nm was recorded.

The glucose-6-phosphatase (G6pase) activity was measured based on the method of Alegre *et al.* [24]. The reaction mixture contained 765 µL of 131.58 mM Hepes-NaOH (pH 6.5), 100 µL of 18 mM EDTA (pH 6.5), 100 µL of 265 mM glucose-6-phosphate, 10 µL of 0.2 M NADP^+^, 0.6 IU/mL mutarotase, and 0.6 IU/mL glucose dehydrogenase. After pre-incubation at 37 °C for 3 min, the mixture was added with 5 µL microsome and incubated at 37 °C for 4 min. The change in absorbance at 340 nm was measured.

The phosphoenolpyruvate carboxykinase (PEPCK) activity was measured using the method of Bentle and Lardy [25]. The reaction mixture contained 72.92 mM sodium Hepes (pH 7.0), 10 mM dithiothreitol, 500 mM NaHCO_3_, 10 mM MnCl_2_, 25 mM NADH, 100 mM IDP, 200 mM PEP, 7.2 unit of malic dehydrogenase, and 10 µL cytosol. The enzyme activity was determined based from the decrease in the absorbance of the mixture at 340 nm at 25 °C.

The superoxide dismutase (SOD) activity was spectrophotometrically measured according to the method of Marklund and Marklund [26]. The SOD was detected based on its ability to inhibit superoxide-mediated reduction. The reaction mixture (50 mM Tris-HCl buffer (pH 8.5), 10 mM EDTA, 0.1 mL cytosol or erythrocyte, and 7.2 mM pyrogallol) was incubated at 25 °C for 10 min and added with 50 µL of 1 N HCl. The absorbance was measured at 420 nm and the activity was expressed as unit/mg protein, wherein one unit represents the amount of enzyme that inhibited the oxidation of pyrogallol by 50%. The amount of protein was determined using Bradford protein assay [27].

The glutathione peroxidase (GPx) activity was measured using the method of Paglia and Valentine [28] with slight modifications. A 0.1 mL of the cytosolic supernatant or erythrocyte was added to the reaction mixture (6 mM glutathione, 1.2 mM NADPH, and 1.25 µM H_2_O_2_ in 20mM Tris-HCl, pH 7.0) that was pre-warmed at 25 °C for 5 min. The mixture was further incubated at 25 °C for 5 min and the absorbance was measured at 340 nm. A molar extinction coefficient of 6.22/mM/cm was used to determine the activity, which was expressed as nmol oxidized NADPH /min/mg protein.

The catalase (CAT) activity was measured according to the method of Aebi [29]. A mixture of 50 mM potassium phosphate buffer (pH 7.4) and 10 µL of mitochondrial fraction or erythrocyte was pre-incubated at 25 °C for 5 min and added with 0.1 mL of 30 mM H_2_O_2_. The disappearance of hydrogen peroxide was monitored spectrophotometrically at 240 nm for 5 min. A molar extinction coefficient of 0.041/mM/cm was used to determine the CAT activity. The activity was defined as the µmol decreased H_2_O_2_/min/mg protein.

The glutathione reductase (GR) activity was determined based on the method of Mize and Langdon [30]. A 10 µL of cytosol or erythrocyte was added to the reaction mixture (1 mM EDTA and 1 mM GSSG in a 0.1 M potassium phosphate buffer, pH 7.4) and the oxidation of NADPH was monitored at 340 nm. The activity was expressed as nmol oxidized NADPH/min/mg protein.

The paraoxonase (PON) activity was measured using the method described by Mackness *et al.* [31]. The microsome or erythrocyte (50 µL) was added to 1 mL Tris/HCl buffer (100 mM, pH 8.0) containing 2 mM CaCl_2_ and 5.5 mM paraoxon. The absorbance of the mixture was measured at 412 nm at 25 °C to determine the generation rate of 4-nitrophenol. The enzymatic activity was calculated using the molar extinction coefficient of 17,100/M/cm.

### 2.8. Statistical Analysis

All data are presented as the mean ± S.E. The data was evaluated by one-way ANOVA using a Statistical Package for Social Sciences software program (SPSS Inc., Chicago, IL, USA) and the differences between the means were assessed using Tukey’s test. Statistical significance was considered at *p* < 0.05.

## 3. Results

### 3.1. Body Weight Gain and Organ Weights

At the end of the experimental period, the high fat-fed mice exhibited markedly higher body weight gain than the control-fed group (Table 3). However, addition of fermented paste in the diet significantly inhibited this high fat-induced body weight gain. The SB and BRG fermented paste, in particular, were able to reduce the weight gain to normal level despite the significant increase in the feed intakes of HF + SB and HF + BRG groups relative to that of the HF mice. The weight of adipose tissues was also substantially higher in HF mice than that of the NC group. On the other hand, all fermented paste-fed groups showed considerably lower adipose tissue weight than the HF mice, with the HF + BRG group having the lowest amount of body fat.

**Table 3:** Body weight gain, feed intake, and adipose tissue weight ^(1)^ in mice fed with high fat diet supplemented with rice fermented paste.

Dietary Group ^(2)^	Body Weight Gain (g)	Total Feed Intake (g)	Adipose Tissue Weight (g)
N	10.72 ± 0.47 ^a^	180.19 ± 2.14 ^a,b^	8.35 ± 0.09 ^a^
HF	17.67 ± 0.46 ^d^	178.54 ± 2.96 ^a^	12.46 ± 0.18 ^c^
HF + SB	11.46 ± 0.87 ^a,b^	184.51 ± 2.75 ^c^	10.94 ± 0.30 ^b^
HF + BR	13.27 ± 1.08 ^c^	184.33 ± 3.28 ^c^	10.50 ± 0.38 ^b^
HF + BRB	12.65 ± 0.39 ^b,c^	181.66 ± 3.90 ^b^	10.33 ± 0.40 ^b^
HF + BRG	10.51 ± 0.10 ^a^	181.37 ± 2.75 ^b^	8.36 ± 0.52 ^a^

^(1)^ Values are mean ± SE (*n* = 8); Means in the same column not sharing a common superscript are significantly different at *p* < 0.05; ^(2)^ NC, normal control AIN76 diet; HF, high fat diet; HF + SB, high fat diet + soybean fermented paste; HF + BR, high fat diet + brown rice fermented paste; HF + BRB, high fat diet + brown rice and rice bran fermented paste; HF + BRG, high fat diet + brown rice and red ginseng marc fermented paste.

### 3.2. Blood Glucose Concentration

**Figure 1:**
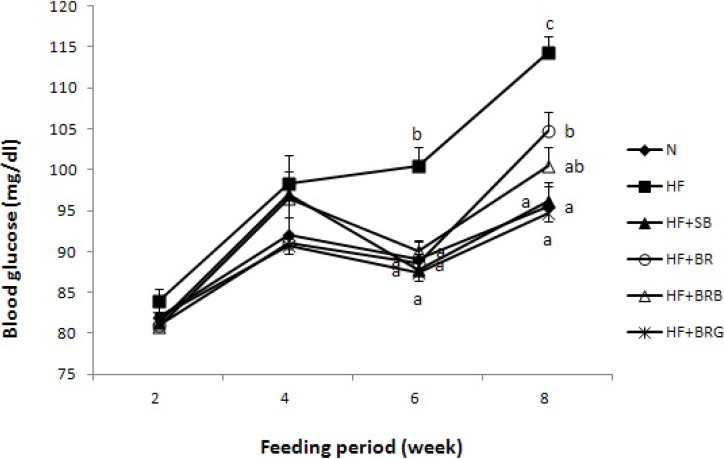
Effect of diet supplementation of rice fermented paste on the blood glucose level in high fat-fed mice. Means not sharing a common superscript are significantly different at *p* < 0.05 (*n* = 8). NC, normal control AIN-76 diet; HF, high fat diet; HF + SB, high fat diet + soybean fermented paste; HF + BR, high fat diet + brown rice fermented paste; HF + BRB, high fat diet + brown rice and rice bran fermented paste; HF + BRG, high fat diet + brown rice and red ginseng marc fermented paste.

The concentration of blood glucose was similar among the animal groups during the second and third weeks of the feeding period (Figure 1). However, on the sixth week, the HF group showed significantly higher glucose level than the other groups. At the end of the experimental period, the HF group exhibited the highest glucose level, followed by the HF + BR group. The addition of SB and BRG fermented pastes in the high fat diet decreased the glucose concentration to normal level.

### 3.3. Glycogen and Insulin Levels

The high fat diet did not significantly change the glycogen concentration, but considerably increased the insulin level (Table 4). Compared with the HF group, all fermented paste-fed groups exhibited substantially higher glycogen concentration and markedly lower insulin level. The HF + BRG mice showed the highest and lowest glycogen and insulin concentrations, respectively, among the animal groups.

**Table 4:** Glycogen and insulin concentrations ^(1)^ in mice fed with high-fat-diet supplemented with rice fermented paste.

Dietary Group ^(2)^	Glycogen (mg/g liver)	Insulin (ng/mL)
NC	4.47 ± 0.48 ^a,b^	8.63 ± 0.30 ^b^
HF	3.60 ± 0.42 ^a^	9.66 ± 0.56 ^c^
HF + SB	5.34 ± 0.35 ^b,c^	8.03 ± 0.61 ^a,b^
HF + BR	4.69 ± 0.66 ^b,c^	8.52 ± 0.74 ^b^
HF + BRB	5.41 ± 0.21 ^c^	8.28 ± 0.42 ^a,b^
HF + BRG	6.50 ± 0.78 ^d^	7.69 ± 0.22 ^a^

^(1)^ Values are mean ± SE (*n* = 8); Means in the same column not sharing a common superscript are significantly different at *p* < 0.05; ^(2)^ NC, normal control AIN76 diet; HF, high fat diet; HF + SB, high fat diet + soybean fermented paste; HF + BR, high fat diet + brown rice fermented paste; HF + BRB, high fat diet + brown rice and rice bran fermented paste; HF + BRG, high fat diet + brown rice and red ginseng marc fermented paste.

### 3.4. Hepatic Glucose-Regulating Enzyme Activities

Significantly higher activity of GK enzyme was found in HF+BRG groups than those of the other groups (Table 5). The G6pase and PEPCK activities significantly increased in HF mice relative to the control group, but fermented paste supplementation decreased the G6pase activity. Moreover, the PEPCK activity was markedly lower in HF + SB and HF + BRG groups compared to that of the HF group.

**Table 5:** Hepatic glucose-regulating enzyme activity in mice fed with high fat diet supplemented with fermented rice paste.

Dietary Group ^(2)^	Enzyme Activity (nmol/min/mg Protein) ^(1)^		
	GK	G6pase	PEPCK
NC	2.49 ± 0.07 ^a^	135.77 ± 0.72 ^c^	2.73 ± 0.06 ^a,b^
HF	2.34 ± 0.08 ^a^	145.68 ± 9.36 ^d^	3.75 ± 0.19 ^c^
HF + SB	2.67 ± 0.21 ^a^	122.04 ± 3.09 ^a^	2.72 ± 0.03 ^a,b^
HF + BR	2.44 ± 0.10 ^a^	131.55 ± 2.90 ^b^	3.19 ± 0.24 ^a,b,c^
HF + BRB	2.71 ± 0.19 ^a^	123.52 ±1.10 ^a^	3.35 ± 0.15 ^b,c^
HF + BRG	3.34 ± 0.19 ^b^	121.80 ± 3.43 ^a^	2.60 ± 0.11 ^a^

^(1)^ Values are mean ± SE (*n* = 8); Means in the same column not sharing a common superscript are significantly different at *p* < 0.05; ^(2)^ NC, normal control AIN76 diet; HF, high fat diet; HF + SB, high fat diet + soybean fermented paste; HF + BR, high fat diet + brown rice fermented paste; HF + BRB, high fat diet + brown rice and rice bran fermented paste; HF + BRG, high fat diet + brown rice and red ginseng marc fermented paste.

### 3.5. Plasma and Erythrocyte Lipid Peroxides

The analysis of TBARS concentration has been widely used to determine the lipid peroxidation and oxidative stress in laboratory animals. In the present study, the levels of plasma and erythrocyte TBARS markedly decreased in fermented paste-fed groups relative to that of the HF group (Table 6). The lowest TBARS concentrations were observed in HF + SB and HF + BRG groups.

**Table 6:** Plasma and erythrocyte TBARS levels ^(1)^ in mice fed with high fat diet supplemented with rice fermented paste.

Dietary Group ^(2)^	Plasma TBARS (nmol/mL)	Erythrocyte TBARS (nmol/g Hb)
NC	13.38 ± 0.48 ^c,d^	3.09 ± 0.10 ^b^
HF	13.68 ± 0.50 ^d^	4.00 ± 0.20 ^c^
HF + SB	12.47 ± 0.25 ^b^	2.08 ± 0.06 ^a^
HF + BR	12.54 ± 0.16 ^b,c^	2.34 ± 0.07 ^a^
HF + BRB	12.62 ± 0.34 ^b,c^	2.44 ± 0.04 ^a^
HF + BRG	11.73 ± 0.57 ^a^	2.04 ± 0.12 ^a^

^(1)^ Values are mean ± SE (*n* = 8); Means in the same column not sharing a common superscript are significantly different at *p* < 0.05; ^(2)^ NC, normal control AIN76 diet; HF, high fat diet; HF + SB, high fat diet + soybean fermented paste; HF + BR, high fat diet + brown rice fermented paste; HF + BRB, high fat diet + brown rice and rice bran fermented paste; HF + BRG, high fat diet + brown rice and red ginseng marc fermented paste.

### 3.6. Hepatic Antioxidant Enzyme Activities

High fat feeding resulted in a significant reduction in the activities of SOD, GR, and PON enzymes (Table 7). However, diet supplementation of fermented paste counteracted the decline in the enzyme activities. Although the GSH-Px and CAT activities were not significantly affected by high fat feeding, they considerably increased with the addition of fermented pastes in the high fat diet.

**Table 7:** Hepatic antioxidant enzyme activity ^(1)^ in mice fed with high fat diet supplemented with rice fermented paste.

Dietary Group ^(2)^	SOD (unit/mg Protein)	GSH-Px (nmol/min/mg Protein)	CAT (µmol/min/mg Protein)	GR (nmol/min/mg Protein)	PON (nmol/min/mg Protein)
NC	1.73 ± 0.10 ^b^	2.18 ± 0.03 ^a^	0.82 ± 0.01 ^a^	14.41 ± 0.22 ^b^	3.46 ± 0.30 ^b^
HF	1.56 ± 0.08 ^a^	2.19 ± 0.04 ^a^	0.80 ±0.02 ^a^	12.48 ± 1.04 ^a^	3.16 ± 0.33 ^a^
HF + SB	2.13 ± 0.42 ^c^	2.87 ± 0.08 ^b^	0.88 ± 0.00 ^b^	16.56 ± 0.71 ^e^	3.62 ± 0.33 ^b,c^
HF + BR	2.16 ± 0.11 ^c^	2.80 ± 0.09 ^b^	0.86 ± 0.00 ^b^	15.13 ± 0.37 ^c^	3.66 ± 0.08 ^c^
HF + BRB	2.55 ± 0.12 ^d^	2.81 ± 0.06 ^b^	0.88 ± 0.01 ^b^	15.48 ± 0.67 ^d^	3.63 ± 0.17 ^b,c^
HF + BRG	2.67 ± 0.08 ^d^	2.94 ± 0.09 ^b^	0.94 ± 0.02 ^c^	17.94 ± 0.19 ^f^	3.75 ± 0.22 ^c^

^(1)^ Values are mean ± SE (*n* = 8); Means in the same column not sharing a common superscript are significantly different at *p* < 0.05; ^(2)^ NC, normal control AIN76 diet; HF, high fat diet; HF + SB, high fat diet + soybean fermented paste; HF + BR, high fat diet + brown rice fermented paste; HF + BRB, high fat diet + brown rice and rice bran fermented paste; HF + BRG, high fat diet + brown rice and red ginseng marc fermented paste.

## 4. Discussion

In the present study, fermented pastes were prepared from soybean, brown rice, or brown rice in combination with rice bran or red ginseng marc. Their comparative effects on the glucose metabolism and antioxidative defense system in high fat-fed mice were investigated. The results showed that fermented paste could effectively reduce the body weight and body fat, improve the glucose metabolism, and enhance the antioxidant defense status in mice under high fat diet condition. While the BR fermented paste significantly decreased the weight gain and adipose tissue weight, addition of red ginseng marc could further enhance its body weight-lowering effects. The antidiabetic effect of fermented soybean in both humans and animal models has been previously reported [32,33]. The increased content of soy peptides and isoflavonoid aglycones during fermentation of soybean is believed to be responsible for the antidiabetic effect of fermented paste and other fermented soybean products [1]. Results of the present study provide the first evidence of the hypoglycemic effect of fermented paste made from brown rice and in combination with rice bran or red ginseng marc. Brown rice contains various bioactive compounds such as gamma oryzanol, gamma aminobutyric acid, ferulic acid, and phytic acid which have been found to have hypoglycemic and antioxidative effects [8,34,35]. It appears that although the BR fermented paste was able to reduce the glucose level in high fat-fed mice, its hypoglycemic effect became more powerful with the inclusion of red ginseng marc. Moreover, the increase in glycogen synthesis and reduction in insulin in HF + SB and HF + BRG groups relative to the HF mice have led to an improved glycemic control in these animal groups. These findings further showed that dietary feeding of BRG fermented paste could improve the glucose metabolism in high fat-fed mice and its hypoglycemic effect was comparable with that of the soybean fermented paste. Park *et al.* [15] previously investigated the effects of SB, BR, BRB, and BRG fermented pastes on the body weight and lipid metabolism in high fat diet-fed mice. Their results also showed that while BR fermented paste effectively reduced the body weight gain and improve the lipid profile in the animals, the addition of red ginseng marc significantly enhanced its body weight-lowering effect and hypolipidemic activity that were comparable to those of the SB fermented paste.

Red ginseng marc is a by-product of ginseng extract production and commonly discarded as waste. Past studies have shown that it contain lipid-soluble components that have biological activities [9,10]. In addition, fermented red ginseng marc was found to have biologically active compounds with strong antioxidant effect [14]. A study conducted by Kim *et al.* [36] showed that fermented red ginseng extract significantly decreased the blood glucose levels in diabetic rats. It was suggested that the enhanced antidiabetic activity of the fermented red ginseng was due to its greater number of ginsenoside types compared with the non-fermented one. Further studies are needed on the identification and characterization of the active components in BR and BRG fermented pastes that have beneficial roles in glucose metabolism for a better understanding of their therapeutic potential against high fat-induced hyperglycemia. The decrease in blood glucose level and enhanced rate of glycogenesis in fermented paste-fed mice, particularly the HF+BRG group, were probably associated with the enhanced activity of the GK enzyme and inhibition of the G6pase and PEPCK activities in the liver. The hepatic enzyme GK is related to glucose homeostasis and its increased expression could cause an increase in blood glucose utilization for energy production or glycogen storage in the liver, leading to a reduction in the blood glucose level [37,38]. The increased rate of glycogenesis found in fermented paste-fed groups, as manifested by an increase in their hepatic glycogen levels, was likely associated with the enhanced GK activity. On the contrary, the enzymes G6pase and PEPCK are associated with gluconeogenesis and hepatic glucose output and their increased activities denote increased glucose level [39,40]. The reduced activities of these enzymes in mice fed with fermented pastes, particularly BRG, may account for the reduction in the blood glucose levels observed in these animals. While the results indicate that both the BR and BRB fermented pastes have significant effect on the glucose metabolism, it seems that the fermented red ginseng marc may have greater hypoglycemic effect than the fermented brown rice.

Oxidative stress is considered the key factor in the pathogenesis of diabetes and its associated metabolic disorders. In the past, various fermented soybean products have been shown to inhibit lipid peroxidation and prevent cellular oxidative damage [16,41,42]. It was found that peptides and isoflavones, particularly genistein and daidzein, have high antioxidative effects [16]. Fermented brown rice and rice bran were also reported to exhibit strong radical scavenging activity [13]. Kim *et al.* [43] accounted that fermented red ginseng extracts were able to reduce the lipid peroxidation in diabetic rats. The present study showed, for the first time, the antioxidative effect of fermented paste made from brown rice. Compared with the SB fermented paste, the BR and BRB fermented pastes seemed to have relatively similar antioxidant potential. However, the addition of red ginseng marc in BR fermented paste resulted in a more potent antioxidative activity. The results indicate that brown rice fermented pastes, especially if added with red ginseng marc, may be useful as functional foods that can provide protection against high fat-induced oxidative stress. The protective effect of fermented paste against oxidative stress may have been partly due to a mechanism associated with the enhancement of the antioxidant enzyme activities. The antioxidant enzymes, which catalyze free radicals-quenching reactions, are part of a highly complex antioxidant protection system developed by the cells in order to control the destructive potential of free radicals and regulate oxidative stress. The enzyme SOD converts superoxide radicals into hydrogen peroxides, which in turn are degraded by the enzymes CAT and GSH-Px into non-toxic products [44]. On the other hand, the GR enzyme converts oxidized glutathione into antioxidant-reduced glutathione and the PON enzyme hydrolyzes oxidized phospholipids and destroys lipid hydrogen peroxides [45,46]. The development of hyperglycemia has been associated with oxidative stress, which results from enhanced production of free radicals and impaired antioxidant defense mechanism [47]. The decreased lipid peroxidation and enhanced activities of the antioxidant enzymes in fermented paste-fed groups indicate a marked improvement in the antioxidant defense system of the animals. In general, the HF+BRG group showed greater enzyme activities than the other groups, which further suggests that the combination of brown rice and red ginseng marc may be valuable in the development of fermented paste with strong antioxidative property.

## 5. Conclusions

The results demonstrate that dietary feeding of fermented paste significantly improved the glucose metabolism and suppressed the oxidative stress in mice under high fat diet condition through a mechanism involving the regulation of the glucose-regulating enzyme activities and activation of the antioxidant enzymes. In general, while the BR and BRB fermented pastes exhibited glucose-lowering action and antioxidative effect, the inclusion of red ginseng marc in brown rice fermented paste markedly enhanced its hypoglycemic activity and antioxidative property that were comparable to that of soybean fermented paste. Brown rice, in combination with red ginseng marc, may be beneficial as a biomaterial in the development of fermented paste with preventive effect against high fat-induced hyperglycemia and oxidative stress.

## References

[1] Kwon D.Y., Daily J.W., Kim H.J., Park S. (2010). Antidiabetic effects of fermented soybean products on type 2 diabetes. Nutr. Res..

[2] Lee G.I., Lee H.M., Lee C.H. (2012). Food safety issues in industrialization of traditional Korean foods. Food Control..

[3] Park K.Y., Jung K.O., Rhee S.H., Choi Y.H. (2003). Antimutagenic effects of doenjang (Korean fermented soypaste) and its active compounds. Mutat. Res..

[4] Jung O.K., Park S.Y., Park K.Y. (2006). Longer aging time increases the anticancer and antimetastatic properties of *doenjang*. Nutrition.

[5] Chai C., Ju H.K., Kim S.C., Park J.H., Lim J., Kwon S.W., Lee J. (2012). Determination of bioactive compounds in fermented soybean products using GC/MS and further investigation of correlation of their bioactive activities. J. Chromatogr. B.

[6] Cicero A.F.G., Derosa G. (2005). Rice bran and its main components: Potential role in the management of coronary risk factors. Curr. Top. Nutraceutical Res..

[7] Kang M.Y., Kim S.M., Rico C.W., Lee S.C. (2012). Hypolipidemic and antioxidative effects of rice bran and phytic acid in high fat-fed mice. Food Sci. Biotechnol..

[8] Kim S.M., Rico C.W., Lee S.C., Kang M.Y. (2010). Modulatory effect of rice bran and phytic acid on glucose metabolism in high fat-fed C57BL/6N mice. J. Clin. Biochem. Nutr..

[9] Lee S.D., Yoo G., Chae H.J., In M.J., Oh N.S., Hwang Y.K., Hwang Y.K., Hwang W.I. (2009). Lipid-soluble extracts as the main source of anticancer activity in ginseng and ginseng marc. J. Am. Oil Chem. Soc..

[10] Lim T.S., Na K., Choi E.M., Chung J.Y., Hwang J.K. (2004). Immunomodulating activities of polysaccharides isolated from Panax ginseng. J. Med. Food.

[11] Katyama M., Yoshimi N., Yamada Y., Sakata K., Kuno T., Yoshida K., Qiao Z., Vihn P.Q., Iwasaki T., Kobayashi H. (2002). Preventive effect of fermented brown rice and rice bran against colon carcinogenesis in male F344 rats. Oncol. Rep..

[12] Kuno T., Hirose Y., Hata K., Kato K., Qiang S.H., Kitaori N., Hara A., Iwasaki T., Yoshimura T., Wada K. (2004). Preventive effect of fermented brown rice and rice bran on *N*-nitrosomethylbenzylamine-induced esophageal tumorigenesis in rats. Int. J. Oncol..

[13] Tomita H., Kuno T., Yamada Y., Oyama T., Asano N., Miyazaki Y., Baba S., Taguchi A., Hara A., Iwasaki T. (2008). Preventive effect of fermented brown rice and rice bran on *N*-methyl-*N*’-nitro-*N*-nitrosoguanidine-induced gastric carcinogenesis in rats. Oncol. Rep..

[14] Jung H.W., Kim J.E., Seo J.H., Lee S.P. (2010). Physicochemical and antioxidant properties of red ginseng marc fermented by *Bacillus subtilis* HA with mugwort powder addition. J. Korean Soc. Food Sci. Nutr..

[15] Park N.Y., Rico C.W., Lee S.C., Kang M.Y. (2012). Comparative effects of doenjang from soybean and brown rice on the body weight and lipid metabolism in high fat-fed mice. J. Clin. Biochem. Nutr..

[16] Kim N.Y., Song E.J., Kwon D.Y., Kim H.P., Heo M.Y. (2008). Antioxidant and antigenotoxic activities of Korean fermented soybean. Food Chem. Toxicol..

[17] Park K.Y., Hwang K.M., Jung K.O., Lee K.B. (2002). Studies on the standardization of doenjang (Korean soybean paste) 1. Standardization of manufacturing method of doenjang by literatures. J. Korean Soc. Nutr..

[18] American Institute of Nutrition (1977). Report of ad hoc committee on standards for nutritional studies. J. Nutr..

[19] McCord J.M., Fridovich I. (1969). Superoxide dismutase: An enzymic function for erythrocuprein (hemocuprein). J. Biol. Chem..

[20] Seifter S., Dayton S., Navic B., Muntwyler E. (1950). The estimation of glycogen with the anthrone reagent. Arch. Biochem..

[21] Ohkawa H., Ohishi N., Yagi K. (1979). Assay for lipid peroxides in animal tissues by thiobarbituric acid reaction. Anal. Biochem..

[22] Hulcher F.H., Oleson W.H. (1973). Simplified spectrophotometric assay for microsomal 3-hydroxy-3-methylglutaryl CoA reductase by measurement of coenzyme A. J. Lipid Res..

[23] Davidson A.L., Arion W.J. (1987). Factors underlying significant underestimations of glucokinase activity in crude liver extracts: Physiological implications of higher cellular activity. Arch. Biochem. Biophys..

[24] Alegre M., Ciudad C.J., Fillat C., Guinovart J.J. (1988). Determination of glucose-6-phosphatase activity using the glucose dehydrogenase-coupled reaction. Anal. Biochem..

[25] Bentle L.A., Lardy H.A. (1976). Interaction of anions and divalent metal ions with phosphoenolpyruvate carboxykinase. J. Biol. Chem..

[26] Marklund S., Marklund G. (1974). Involvement of the superoxide anion radical in the autoxidation of pyrogallol and convenient assay for superoxide dismutase. Eur. J. Biochem..

[27] Bradford M.M. (1976). A rapid sensitive method for the quantitation of microgram quantities of protein utilizing the principle of protein-dye binding. Anal. Biochem..

[28] Paglia E.D., Valentine W.N. (1967). Studies on quantitative and qualitative characterization of erythrocyte glutathione peroxidase. J. Lab. Clin. Med..

[29] Aebi H., Bergmeyer H.U. (1974). Catalase. Method of Enzymatic Analysis.

[30] Mize C.E., Langdon R.G. (1952). Hepatic glutathione reductase, purification and general kinetic properties. J. Biol. Chem..

[31] Mackness M.I., Arrol S., Durrington P.N. (1991). Paraoxonase prevents accumulation of lipoperoxides in low-density lipoprotein. FEBS Lett..

[32] Fujita H., Yamagami T., Oshima K. (2001). Long-term ingestion of a fermented soybean-derived Touchi-extract with alpha-glucosidase inhibitory activity is safe and effective in humans with borderline and mild type-2 diabetes. J. Nutr..

[33] Fujita H., Yamagami T. (2001). Fermented soybean-derived Touchi-extract with anti-diabetic effect via alpha-glucosidase inhibitory action in a long-term administration study with KKAy mice. Life Sci..

[34] Purwana I., Zheng J., Li X., Deurloo M., Son D.O., Zhang Z., Liang C., Shen E., Tadkase A., Feng Z.P. (2014). GABA promotes human β-cell proliferation and modulates glucose homeostasis. Diabetes.

[35] Son M.J., Rico C.W., Nam S.H., Kang M.Y. (2011). Effect of oryzanol and ferulic acid on the glucose metabolism of mice fed with a high-fat diet. J. Food Sci..

[36] Kim H.J., Chae I.G., Lee S.G., Jeong H.J., Lee E.J., Lee I.S. (2010). Effects of fermented red ginseng extracts on hyperglycemia in streptozotocin-induced diabetic rats. J. Ginseng Res..

[37] Coope G.J., Atkinson A.M., Allott C., McKerrecher D., Johnstone C., Pike K.G., Holme P.C., Vertigan H., Gill D., Coghlan M.P. (2006). Predictive blood glucose lowering efficacy by glucokinase activators in high fat fed female zucker rats. Br. J. Pharmacol..

[38] Ferre T., Riu E., Bosch F., Valera A. (1996). Evidence from transgenic mice that glucokinase is rate limiting for glucose utilization in the liver. FASEB J..

[39] She P., Shiota M., Shelton K.D., Chalkley R., Postic C., Magnuson M.A. (2000). Phosphoenolpyruvate carboxykinase is necessary for the integration of hepatic energy metabolism. Mol. Cell. Biol..

[40] Van Schaftingen E., Gerin I. (2002). The glucose-6-phosphatase system. Biochem. J..

[41] Wang D., Wang L.J., Zhu F.X., Zhu J.Y., Chen X.D., Zou L., Saito M., Li L. (2008). *In vitro* and *in vivo* studies on the antioxidant activities of the aqueous extracts of Douchi (a traditional Chinese salt-fermented soybean food). Food Chem..

[42] Ahn C.B., Je J.Y. (2011). Antioxidant activity of traditional Korean fermented soybean (damdusi) extract on free radical-mediated oxidative systems. J. Food Biochem..

[43] Kim H.J., Lee S.G., Chae I.G., Kim M.J., Im N.K., Yu M.H., Lee E.J., Lee I.S. (2011). Antioxidant effects of fermented red ginseng extracts in streptozotocin-induced diabetic rats. J. Ginseng Res..

[44] Reiter R.J., Tan D., Burkhardt S. (2002). Reactive oxygen and nitrogen species and cellular and organismal decline: Amelioration with melatonin. Mech. Aging Dev..

[45] Mullineaux P.M., Creissen G.P., Scandalios J.G. (1997). Glutathione reductase: Regulation and role in oxidative stress. Oxidative Stress and the Molecular Biology of Antioxidant Defenses.

[46] Ng C.J., Shih D.M., Hama S.Y., Villa N., Navab M., Reddy S.T. (2005). The paraoxonase gene family and atherosclerosis. Free Radic. Biol. Med..

[47] Maritim A.C., Sanders R.A., Watkins J.B. (2003). Diabetes, oxidative stress, and antioxidants: A review. J. Biochem. Mol. Toxicol..

